# *Aulacaspis yasumatsui* Invasion Reduced *Cycas micronesica* Microstrobilus Size and Pollinator Brood Site Competence

**DOI:** 10.3390/insects12111023

**Published:** 2021-11-13

**Authors:** Thomas E. Marler, L. Irene Terry

**Affiliations:** 1 Western Pacific Tropical Research Center, University of Guam, Mangilao, GU 96923, USA; 2 School of Biological Sciences, University of Utah, Salt Lake City, UT 84112, USA; irene.terry@utah.edu

**Keywords:** *Anatrachyntis*, brood site pollination, coextinction, invasive species

## Abstract

**Simple Summary:**

The armored scale known as cycad aulacaspis scale invaded Guam in 2003 and began killing individuals of the host tree *Cycas micronesica* by 2005. The plant mortality dynamics of this case study have been reported in detail. However, the influence of this invasion on reproductive behaviors of the host tree has not been reported. We took a look at how the male cones of this gymnosperm tree were influenced by the invasion over many years. As expected, male cones decreased substantially in size after the invasion, but then began recovering shortly thereafter. As of 2021, the cones remain only 57% as large as the pre-invasion cones. The mutualist pollinator relies on these cones as brood sites, and the ability of male cones to produce offspring of the mutualist pollinator was similarly reduced by the invasion. Conservationists need new knowledge such as this to inform species recovery efforts.

**Abstract:**

*Aulacaspis yasumatsui* Takagi invaded Guam in 2003, and the influence on survival and demography of the host *Cycas micronesica* K.D. Hill population has been well-studied. To more fully understand how *A. yasumatsui* has threatened the host cycad species, we determined the microstrobilus size and number of pollinators per microstrobilus from 2001 to 2021. The microstrobilus height and diameter were measured directly, and the volume was calculated. Microstrobili were 58 cm in height, 13 cm in diameter, and 4740 cm^3^ in volume prior to direct *A. yasumatsui* infestations. Microstrobili decreased in size immediately after direct infestations by *A. yasumatsui*, and then began to slowly increase in size until 2021. For example, the volume was 24% of pre-invasion volume in 2007, and was 57% of pre-invasion volume in 2021. Microstrobili were harvested; then, the number of pollinator pupae were counted after an incubation period. Pollinator pupae counts per microstrobilus declined to 66% of pre-invasion levels by 2007 and have remained similarly constrained through 2021. Our results revealed that *A. yasumatsui* damage to the host *C. micronesica* population is not limited to attrition of the extant plant population, but also includes a loss in male reproductive effort and the risk of coextinction of the insular pollinator.

## 1. Introduction

The invasion of Guam by *A. yasumatsui* was documented in 2003 [1,2], and the resulting in situ *C. micronesica* plant mortality was recognized by 2005 [3]. The projected plant mortality as a result of this invasion was the basis of an endangered listing by 2006 [4]. This arborescent cycad species was the most abundant tree on Guam at the time of the invasion [5], but the mortality due to a coalition of biotic threats reached 96% by 2020 [6].

The infestations of this nonnative specialist armored scale have generated numerous negative outcomes at the organismal and population levels. For example, nonstructural carbohydrate concentration [7] and carbon dioxide efflux [8] from stems declined following *A. yasumatsui* infestations. Leaf infestations changed leaflet chemistry in a manner that predicted more rapid litter decomposition and nutrient turnover following *A. yasumatsui* herbivory [9]. The tree’s intrinsic resistance to tropical cyclone damage was impaired by the invasion, leading to increased tree mortality during tropical cyclones [10,11,12].

Cycad plants depend on insect pollinator mutualists, many of which exploit the microstrobilus tissues as brood sites for regeneration [13]. Several insects are associated with *C. micronesica* microstrobili [1,14]. The moth *Anatrachyntis* Meyrick is the only one of these species known to exploit the microstrobilus tissue as a brood site. Additionally, the adults visit but do not feed on the megastrobilus tissues on female trees. The rapid removal of microstrobilus tissues from the male trees by the larvae of this moth has been shown to increase male tree fecundity by shortening the time between successive reproductive events [15].

In situ *C. micronesica* seedlings comprised the first demographic group to be removed from Guam’s forests by the *A. yasumatsui* infestations [3]. When we noticed that the regeneration of the plant population was no longer replacing the killed seedlings, we predicted several mechanisms through which the armored scale behaviors may influence reproductive biology in a manner that exacerbated the extinction threats [16]. To our knowledge, no reports have included results from experimental or observational studies addressing the influence of the *A. yasumatsui* invasion on the reproductive biology of *C. micronesica*. Our objectives were to follow through on our predictions by quantifying the long-term changes in microstrobili size and the brood site competence of the microstrobili on the east coast karst forests in northern Guam.

## 2. Materials and Methods

Our field work began in June 2001 and continued through March 2021. The peak season for microstrobili emergence was April–May [17], with pollen dehiscence occurring in June–July. The field measurements and strobilus collections were accomplished during June–July of each year for 2001–2005. The habitats were invaded by *A. yasumatsui* in late 2005, and the scale predator *Rhyzobius lophanthae* Blaisdell was purposefully released at the same time. The scale population irruptions were more pronounced initially than the *R. lophanthae* populations were. Therefore, we found no microstrobili that were adequately protected from direct *A. yasumatsui* infestations throughout 2006, so there were no data for that year. From 2007 until 2021, reproductive events were minimal during most of the year, so we did not restrict the field work to any particular season.

The footprint of the forests from which microstrobili were measured and collected was the northeast coast of Guam. The latitudinal range was 13.468845°–13.548023°, the longitudinal range was 144.853037°–144.932938°, and the elevational range was 50–150 m above sea level. This terrain contained high-density areas of occupancy in 2001 with the *C. micronesica* populations represented by numerous individuals of all size categories from seedlings and juveniles up to trees with stems of 5+ m.

**Figure 1:**
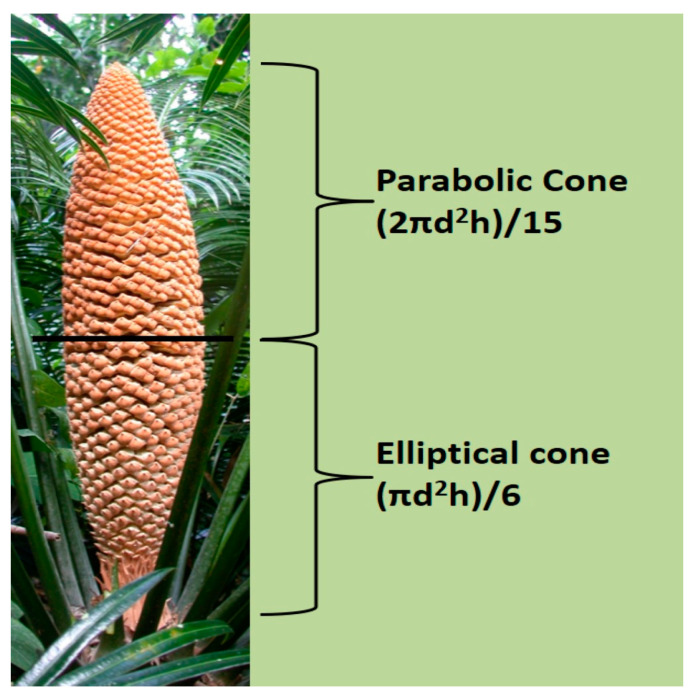
The volume of each *Cycas micronesica* microstrobilus was calculated by assigning the formula for the parabolic cone to the apical half and the formula for the elliptical cone for the basal half. d = maximum diameter, h = height.

A total of 50 microstrobili were measured each calendar year to quantify mean height and diameter. The stem height was measured from the soil surface to the top of the cataphylls that terminate the stem apex. The maximum height of a microstrobilus occurs immediately after pollen dispersal when the central axis has fully expanded [18]. The megastrobili that were measured were restricted to this stage. For each replication, the microstrobilus volume was calculated for the apical half using the formula for a parabolic cone and for the basal half using the formula for an elliptical cone (Figure 1) [19]. The total microstrobilus volume was calculated by adding the volume of the apical and basal halves.

Methods to accurately count the number of *Anatrachyntis* individuals within each microstrobilus were developed in 2005 through trial and error. The first requirement was to determine a standardized phase for harvesting microstrobili. *Anatrachyntis* adults enter the microstrobili for the social relations of sexual encounters and ovipositing during the pollen dispersal stage. We restricted our choice of microstrobili to those with apical pollen sacs fully open and fresh pollen readily visible on the adaxial surfaces of apical sporophylls, the stage that signifies termination of pollen dispersal. We have not conducted extensive ethology studies with this moth, and ovipositing may continue after this stage. Therefore, our population count data may be slight underestimations. However, the same methods were employed for every year, so the possible underestimation would be homogeneous among all of the years in our study.

The first attempt at counting pollinator individuals employed an intact microstrobilus harvested at the end of the pollen dispersal period. The microstrobilus was placed in a screen cage and incubated through the larvae and pupae stages, and the resulting pollinator adults emerged from the tissue. However, the adults were highly mobile and the numbers of individuals were excessive, so no method to accurately count the adults was perceivable without euthanizing them. This method was not accepted. The second attempt was to cut the microstrobilus into six equal vertical sections to reduce the number of adults in each of the six cages for each microstrobilus. Accurately counting the number of adults in each cage was still an unmanageable task due to the excessive counts, so this method was not accepted. Finally, a method to count the pupae rather than the adults was developed. Each harvested microstrobilus was transported to an ambient laboratory and cut into six equidistant vertical sections. The sections were placed in six separate cages that were 0.072 m^3^ and made of white polyester fabric with 130 holes per centimeter. The cages were monitored several times per day until the initial *Anatrachyntis* adult was found in one of the six cages. The tissue and frass were carefully teased apart to find all of the remaining pupae, and the total number of pupae within each cage was counted directly. Thereafter, the pupae, frass, and tissue were combined into one cage and then transported back to the habitat from which the microstrobilus had been harvested, where they were poured into the crowns of several *C. micronesica* trees to provide an opportunity for the adults to emerge from the pupae in their habitat of origin. Locating microstrobili at the extreme end of the pollen dispersal stage proved difficult during our stochastic field visits in the post-invasion years. Therefore, we standardized this method to locate six microstrobili for each of the years 2005, 2008, 2011, 2014, 2018, and 2021. Stem height and microstrobilus height and diameter were measured for each of the six trees. The relative volume of microstrobilus tissue that generated each pupa was calculated by dividing the volume of each strobilus by the total number of pupae.

Statistical analyses were performed using R (Version 3.6.3). To determine differences between the pre-invasion years and the post-invasion years for the variables microstrobilus height, diameter, and volume, we performed *t*-tests after checking for data normality (Shapiro–Wilk test) and homogeneity of variances (Levene’s test, across the two groups). All data for strobilus and stem height measurements indicated a normal distribution (Shapiro–Wilk test, *W* > 0.91, *p* > 0.27), but variances were not equal for some measurements (Levene’s Test, *F*_1,18_ = 4, *p* < 0.048). For those measurements, a Welch–Satterthwaite *t*-test was performed that adjusts for unequal variance. Otherwise, a Student’s *t*-test was used. To examine trends during the post-invasion years, a regression analysis tested the effect of year on each of these microstrobilus measurements. A Student’s *t*-test (variances were equal: Levene’s test, *F*_1,8_ = 3.64, *p* = 0.093) was conducted to compare the last five years of post-invasion (2017–2021) values with those of the pre-invasion benchmark years. Finally, we determined the influence of tree height on our response variables using two approaches. First, we used regression analysis with each microstrobilus size metric as the dependent variable and tree height as the independent variable. Second, we conducted an analysis of covariance with year as the main effect and tree height as the covariate. The data for the number of pupae and volume of strobilus per pupa across the 6 years examined were not normally distributed (Shapiro–Wilk test, *W* < 0.86, *p* < 0.0001), and variances were unequal (Levene’s test, *F*_5,30_ > 11, *p* < 0.001), so we performed Kruskal–Wallis Analysis of Variance to examine these variables. There were 50 replications for the microstrobilus and stem size metrics (the same trees were not measured across years), and six replications for the brood site pupae metrics. The stem and microstrobilus size data were not subjected to statistical analyses for the brood site data.

We created scatter plots of the influence of microstrobilus strata on pupae counts for each year and a nonlinear trend characterized the scatter with the median strata exhibiting the greatest number of pupae. Therefore, we fitted the data for each year with a quadratic model.

## 3. Results

### 3.1. Microstrobilus Size

**Figure 2:**
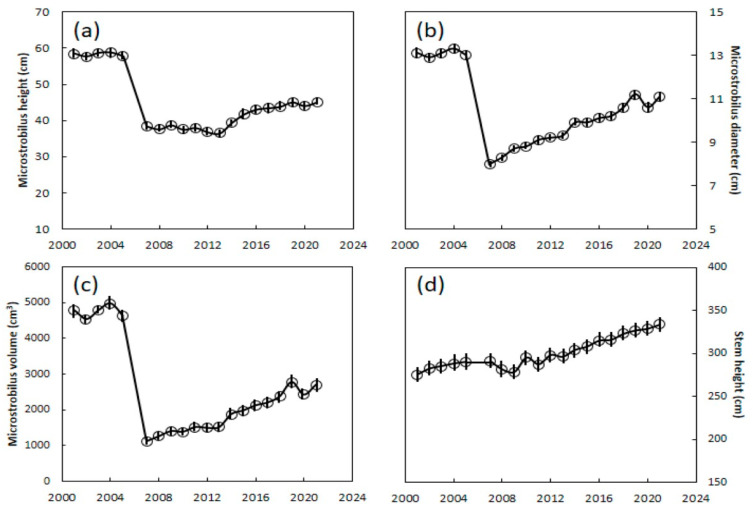
*Cycas micronesica* stem and microstrobilus size in Guam karst forest from 2001 to 2021. (**a**) Microstrobilus height; (**b**) microstrobilus diameter; (**c**) microstrobilus volume; (**d**) stem height. Mean ± SE, *n* = 50.

No significant change occurred in microstrobilus size from 2001 to 2005 (linear regression, with no significant slope coefficients of the three measurements, linear regression, *F*_1,3_ = 0.05 or greater, *p* = 0.71 or greater) and the microstrobili were 58 cm in height, 13 cm in diameter, and 4740 cm^3^ in volume during these years (Figure 2). These metrics represent the benchmark of pre-invasion microstrobilus size, as the *A. yasumatsui* infestations were initiated in this habitat in late 2005. The microstrobilus size was substantially decreased by 2007 as a result of *A. yasumatsui* infestations, when microstrobili were 38 cm in height, 8 cm in diameter, and 1119 cm^3^ in volume. Each of the megastrobilus measurements in the post-invasion years (2007–2021) were significantly smaller than those of the pre-invasion years (Welch–Satterthwaite *t*-test for unequal variances, df = 16 to 18.0, *t* = 12.59 or greater, *p* < 0.0001). There were significant changes across the post-invasion years. The microstrobilus height remained relatively unchanged until 2013 as there was a slight negatively significant slope over those years with a slope coefficient of −0.29 (linear regression, *F*_1,6_ = 7.72, *p* = 0.039, *r*^2^ = 0.56). The height then increased with each year until 2019 with a significantly positive slope coefficient of 1.3 (linear regression, *F*_1,5_ = 40.6, *p* = 0.0014, *r*^2^ = 0.87). The microstrobili height remained stable at about 45 cm for the years 2019–2021. The microstrobilus diameter increased gradually from 2007 until 2019 with a small but significantly positive slope coefficient of 0.244 (linear regression *F*_1,11_ = 387.6, *p* < 0.0001, *r*^2^ = 0.97). The mean microstrobilus diameter was about 11 cm for the years 2019–2021. The changes in microstrobilus volume mirrored those of the diameter, and the volume increased gradually from 2007 until 2019 with a significant positive slope of 122.2 (linear regression, *F*_1,11_ = 149.2, *p* < 0.0001, *r*^2^ = 0.93). The mean microstrobilus volume was about 2630 cm^3^ for the years 2019–2021. Even though each strobilus measurement indicated some increase in size, the last five years of each measurement were significantly smaller than those of the 2001–2005 benchmark years (Student’s *t*-test, df = 8, *t* = 12.1 or greater, *p* < 0.0001).

The stem height of the trees used for microstrobilus measurements remained stable from 2001 until 2012, ranging from 275 cm to 300 cm. Beginning in 2013, the stem height began to increase slowly until reaching a mean of 334 cm in 2021 (Figure 2d), with a significant positive slope during those years, with a regression coefficient of the year on the stem height of 4.5 (linear regression results, *F*_1,7_ = 356.5, *p* < 0.0001, *r*^2^ = 0.98).

Tree height did not exert a direct influence on microstrobilus size. Our regression analysis revealed an inadequate fit of the model for height (*r*^2^ = 0.003), diameter (*r*^2^ = 0.001), or volume (*r*^2^ = 0.005). Our analysis of covariance revealed nonsignificance of the tree height covariate for microstrobilus height (*p* = 0.810), diameter (*p* = 0.116), or volume (*p* = 0.220).

### 3.2. Pollinator Pupae

**Figure 3:**
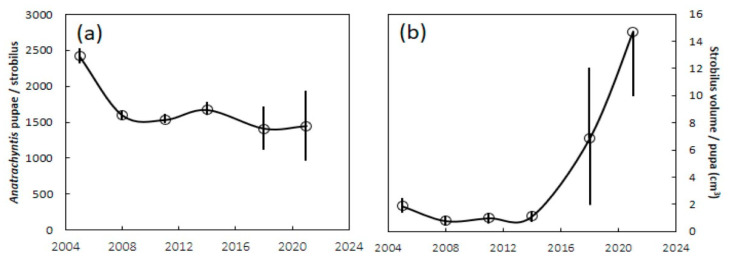
The number of *Anatrachyntis* pupae in *Cycas micronesica* microstrobili in Guam karst forest from 2005 to 2021. (**a**) Pupae per strobilus; (**b**) strobilus volume per pupa. Mean ± SE, *n* = 6.

**Figure 4:**
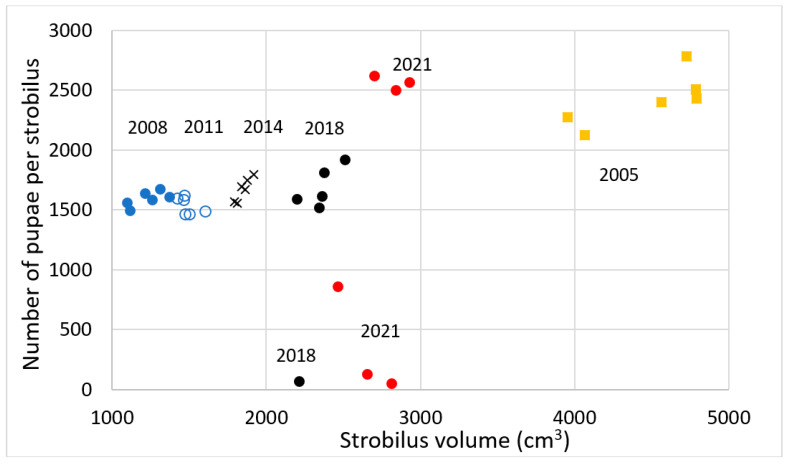
The number of pupae per strobilus versus strobilus volume, with 2005 being the pre-invasion year, and then the post-invasion years starting in 2008 until 2021. Orange squares = 2005 data; blue-filled circles = 2008 data; blue open circles = 2011 data; black ‘x’ = 2014 data; black circles = 2018 data; and red circles = 2021 data.

Guam’s 2005 male *C. micronesica* trees provided a vital ecosystem function by serving as brood sites for the recruitment of 2400+ *Anatrachyntis* individuals per microstrobilus (Figure 3a and Figure 4) The average number of *Anatrachyntis* pupae in *C. micronesica* microstrobili declined to about 1600 individuals immediately after the *A. yasumatsui* invasion, and then did not increase through 2021. However, a nonparametric Kruskal–Wallis one-way analysis of variance indicated that the only years with a significantly smaller number of pupae were in 2008 and 2011 (Kruskall–Wallis Statistic = 12.8, df 5, with P, χ^2^ approximation, = 0.03). The stem height and microstrobilus size metrics were similar to those reported in Figure 2 (see Figure 4 for microstrobilus size for each year).

The efficiency of strobilus tissue in generating pupae was estimated by the volume of strobilus tissue per pupa. This metric declined abruptly immediately after the *A. yasumatsui* invasion (Figure 3b). The average volume of strobilus tissue that generated each pupa remained relatively stable from 2005 until 2014, and then increased dramatically in 2018 and again in 2021. These increases were a result of very few pupae being found in some microstrobili (Figure 4), suggesting declines in pollinator population. Again, a Kruskall–Wallis test indicated that only 2008 and 2011 had less strobilus tissue that generated pupa than in 2005 (Kruskall–Wallis Statistic = 27.9, df 5, with P, χ^2^ approximation = 0.0001).

### 3.3. Stratification

**Figure 5:**
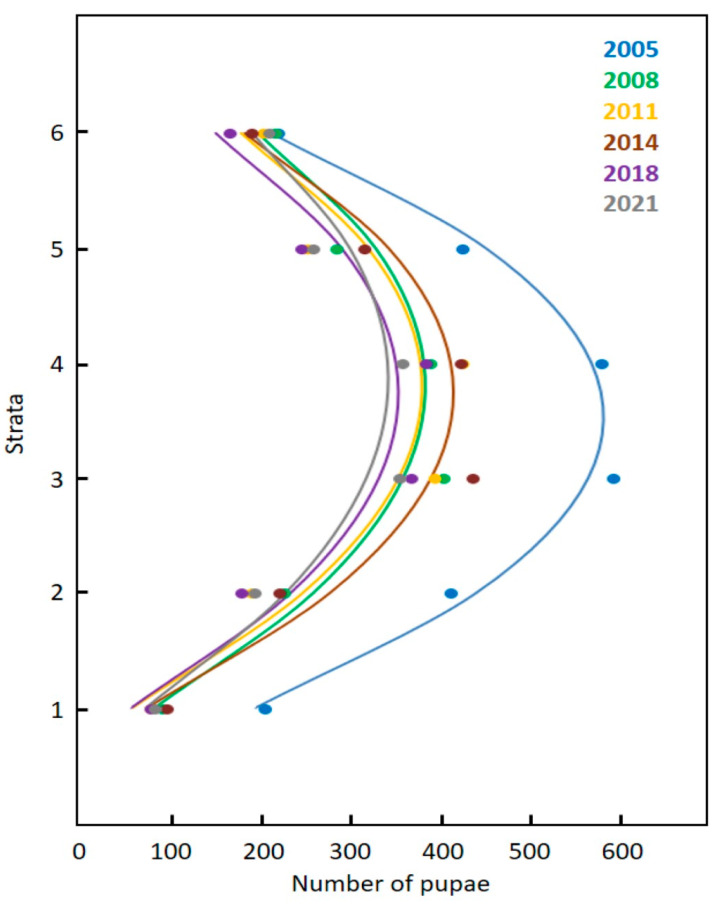
The influence of strata on the number of *Anatrachyntis* pupae in *Cycas micronesica* microstrobili in Guam karst. Forest from 2005 to 2021. Stratum 1 = basal stratum; strata 6 = apical stratum. 2005: pupae = −173 + (427 × strata) − (61 × strata^2^). *r*^2^ = 0.96. 2008: pupae = −171 + (291 × strata) − (38 × strata^2^). *r*^2^ = 0.92. 2011: pupae = −215 + (313 × strata) − (41 × strata^2^). *r*^2^ = 0.85. 2014: pupae = −221 + (339 × strata) − (45 × strata^2^). *r*^2^ = 0.92. 2018: pupae = −201 + (296 × strata) − (40 × strata^2^). *r*^2^ = 0.88. 2021: pupae = −150 + (254 × strata) − (33 × strata^2^). *r*^2^ = 0.90.

A quadratic model characterized the influence of microstrobilus stratification on the number of pupae among the six equidistant sections (Figure 5). For 2005, the number of pupae in the midsection strata was 2.8-fold greater than in the apical and basal strata, and the number of pupae in the two extreme strata were similar. For the remaining years, the midsection strata contained the greatest number of pupae, but there were more pupae in the apical strata than in the basal strata.

## 4. Discussion

The invasion of Guam by *A. yasumatsui* was predicted in 2000 [20], occurred in 2003 [2], and then devastated the population of the native host tree *C. micronesica* [6]. The invasion of this nonnative specialist armored scale has generated numerous negative outcomes at the organismal and population levels [3,6,7,8,9,10,11,12]. Herein, we have expanded the knowledge concerning the influence of this invasion on Guam’s biodiversity by reporting that *C. micronesica* microstrobili exhibited a profound reduction in size and capacity to function as a brood site for *Anatrachyntis* recruitment. Therefore, this invasion has damaged *C. micronesica* reproductive biology in conformity with our earlier prediction [16], outcomes that have not been reported until now.

Our data from 2001–2005 enable an objective understanding of microstrobilus size and brood site competence during the final era of healthy *C. micronesica* populations on Guam, as the *A. yasumatsui* population entered our study site in late 2005. The initial *A. yasumatsui* damage reduced the microstrobilus size to a minimum in 2007, and the size metrics have been slowly returning toward the pre-invasion size through the end of our study. However, as of 2021, the microstrobilus volume remained at only 57% of the pre-invasion volume, signifying a sustained reduction in male tree fitness. A look at the brood site performance revealed a similar decline after the invasion. Prior to the *A. yasumatsui* invasion, *C. micronesica* microstrobili contained 2400+ *Anatrachyntis* pupae. Immediately after the invasion, the number of pupae per microstrobilus was reduced to 66% of the pre-invasion levels. Unfortunately, no recovery in mean number of pupae has occurred even as the microstrobilus size has slowly increased since 2007, signifying a sustained reduction in brood site competence and general declines in pollinator populations. Another indication of pollinator decline is that the 2018 and 2021 sample years had the lowest numbers of pupae in some strobili (Figure 4). We also observed numerous microstrobili beginning in 2006 that were directly infested with *A. yasumatsui*, and in those cases, the microsporophylls did not open adequately to enable pollinator entry. Our data herein did not include any of those infested microstrobili.

How do chronic infestations of an armored scale generate smaller male reproductive structures on host trees? We suggest that the direct influence of the herbivore on nonstructural carbohydrate relations of storage organs may be causal. We have shown that decreases in starch and sugars in *Cycas* organs accompany scale-induced plant mortality [21]. We have also shown that starch and sucrose concentrations decline in *Cycas* stems during the production of microstrobili [22]. Therefore, the chronic infestations of this armored scale likely place the mature plants in a chronic status of limited nonstructural resources, forcing microstrobilus time-size tradeoff decisions by male trees that may be expressed as less frequent or smaller reproductive organs within an optimal-allocation model; see [23]. Within this context, the recent slow recovery of microstrobilus size toward pre-invasion dimensions may be a proxy that indirectly signifies a concomitant slow recovery of nonstructural resources in storage organs.

Cycads are the most threatened plant group studied to date [24]. The threats for most cycad species are habitat loss, habitat degradation, and poaching [25]. However, invasions are becoming more damaging to biodiversity globally [26]. This well-studied example of how an island invasion led to the endangerment of the Guam cycad and probably its mutualist pollinator may foreshadow other cases where similar invasion threats will occur. Indeed, learning more about the Guam case study may prove to be useful in mitigating the damages during these future events [27].

Shortly after *A. yasumatsui* began a series of new country invasions, the International Union for Conservation of Nature Cycad Specialist Group considered *A. yasumatsui* as the single most important threat to natural cycad populations [28]. A national species recovery plan has not been drafted for *C. micronesica* despite the fact that the species has been listed under the United States Endangered Species Act for 6 years [29]. When the formal planning for species recovery begins, our results will aid in conservation decision-making. The enactment of this plan carries the potential to serve as an example for global cycad conservation approaches wherever invasive species are among the threats. For example, *Cycas taitungensis* Shen, Hill, Tsou & Chen is the second cycad species that has been threatened within its endemic range by an *A. yasumatsui* invasion [20]. Although the pollinator of *C. taitungensis* is not known to date, the influence of *A. yasumatsui* on *C. taitungensis* reproductive biology likely has parallels to the Guam case study.

Benchmarking is important in conservation and employs a contemporary or historical point of reference to expand knowledge for improving evidence-informed conservation decisions [30,31,32]. Our 2001–2005 data provide that benchmark for historical microstrobilus size and brood site competence. We propose the use of these objective microstrobilus size and brood site metrics as an accurate approach for conservationists to determine the pace of species recovery in response to future conservation interventions. Height increment has also been reduced in Guam’s *C. micronesica* population as a result of the *A. yasumatsui* invasion, and it has been similarly proposed as an objective measurement of species recovery into the future [33].

To our knowledge, we are the first to take an empirical look at microstrobilus strata in relation to brood site function. We believe the greater number of pupae in the midsections is a direct result of the greater volume of sporophyll tissue for tunneling by the larvae. This can be relatively observed in Figure 1. The relative number of pupae in each stratum may be a function of preferential ovipositing by adults in the midsections. However, the late instar larvae are highly mobile in laboratory incubation studies (personal observations), so the number of pupae within each stratum may also be influenced by larvae mobility prior to pupation. More ethology studies are needed to elucidate the causal mechanisms of the vertical stratification of pollinator pupae.

The brood site competence metrics are also useful for discussing the potential for coextinction [34] of the pollinator. To our knowledge, Guam’s *Anatrachyntis* population is reliant on the availability of *C. micronesica* microstrobilus tissue for larval food, and the potential to lose this moth taxon from the biosphere as the *C. micronesica* population declines is very real. Coextinction is possible but the *Anatrachyntis* species identity is not currently known. Without a definitive identification, this native insect cannot be given the protection that it needs. This should be corrected with a concerted effort to describe this insular moth taxon so the conservation community is fully aware of the full extents of the Guam biodiversity threats.

The increase in tree height throughout the timeline of our study was a result of the preferential culling of smaller individuals by the nonnative insect infestations as the time of infestation progressed [6]. This did not signify that the persisting shorter trees began to refrain from reproducing as time progressed. Contrarily, these results were an indirect quantification of the fact that the persisting trees were among the tallest height categories of the population when the invasion occurred almost 20 years ago. The lack of influence of tree height on microstrobilus size was expected based on previous studies, which indicated that tree height did not influence megastrobilus size [35].

The decline in the mean number of pupae shortly after the invasion was a result of the homogeneous reduction in pupae counts for each microstrobilus. However, in recent years, the reduced mean has been a result of some strobili containing many pupae but other strobili containing few pupae. We have recorded the same trends when trapping adult moths entering microstrobili (unpublished data). These trends may foreshadow the beginning of the pollinator population collapse and indicate that the timing and proximity of nearby microstrobili may exert a direct effect on the number of pupae within each strobilus. *Anatrachyntis* is a cosmopolitan genus that feeds on a wide range of plant hosts [36]. *Anatrachyntis badia* (Hodges), which has a wide range of hosts in the US, has been reported from cones of *Zamia integrifolia* in Florida, USA [37] and from leaves of cultivated *Cycas* species in Italy [38]. Examination of the morphology and molecular data of moths on megastrobili of *C. micronesica* on Yap indicate that it is in the genus *Anatrachyntis* but is a different species (morphological determination by T. Evans, Australian National Insect Collection; molecular phylogenetic determination by S. Salzman, Cornell University). Therefore, the threat of extinction in this case study is actually greater than just the host tree. Indeed, the extirpation of *C. micronesica* on Guam and Rota would lead to extinction of this endemic moth taxon.

This Guam case study may also serve as an example of an invasional meltdown [39]. Indeed, a number of interacting nonnative herbivores have created a coalition of biological threats to this gymnosperm tree [40]. The interactions among some of these herbivores are species-specific [41] and have not been adequately studied to date. We focused on *A. yasumatsui* for this study because it is the only nonnative herbivore that leads to tree mortality. The three nonnative entomological leaf herbivores invaded Guam within the timespan of 2003–2005 [41]. The *Cycas* specialist butterfly *Luthrodes pandava* Horsfield oviposits on young expanding leaves and appears to be in direct competition with *A. yasumatsui*. The leaf miner *Erechthias* sp. Meyrick oviposits on mature leaves, and the incidence is negatively correlated with antecedent *A. yasumatsui* infestations. Our findings indicate that further studies designed to understand the myriad interactions among the herbivores and the threatened native taxa are warranted in order to conserve Guam’s endangered species.

In summary, chronic infestations of invasive insect herbivores on Guam have altered numerous organismal and population traits of the island’s cycad *C. micronesica*. We have shown that the size of strobili produced by male trees and the competence of these strobili to serve as brood sites for the moth pollinator were abruptly decreased shortly after the 2003 invasion of *A. yasumatsui*, the greatest ongoing threat to the plant. As of 2021, the strobilus size has shown some signs of recovery in size, but the number of pollinator pupae generated by each strobilus has not. Continued monitoring of these and other biology and ecology changes will be required to fully understand how Guam’s environment will respond to the global changes that impact this isolated Micronesian island.

## Data Availability

Data available upon request.
